# Assessing *Metschnikowia pulcherrima* as a potential probiotic yeast for animal feed

**DOI:** 10.1093/sumbio/qvae008

**Published:** 2024-04-25

**Authors:** Montazar Al-Nijir, Daniel A Henk, Michael R Bedford, Christopher J Chuck

**Affiliations:** Department of Chemical Engineering, University of Bath, Bath BA2 7AY, United Kingdom; Department of Biology and Biochemistry, University of Bath, Bath BA2 7AY, United Kingdom; Department of Biology and Biochemistry, University of Bath, Bath BA2 7AY, United Kingdom; AB Vista, Woodstock Court, Marlborough SN8 4AN, United Kingdom; Department of Chemical Engineering, University of Bath, Bath BA2 7AY, United Kingdom

## Abstract

In response to escalating antimicrobial resistance fuelled by their extensive use in livestock farming, this study explores alternative strategies in animal health management. We investigated the probiotic potential of *Metschnikowia pulcherrima* strains 4 × 3, DH5, ICS1, and QRI1 for their suitability in poultry environments. Our research demonstrated that previously discovered antimicrobial *M. pulcherrima* strains exhibit promising probiotic behaviours, including biofilm production, auto-aggregation, cell surface hydrophobicity (CSH), adhesion to Caco-2 cells, antioxidant capacity, and resilience to gastric stresses. Notable findings include DH5 exhibiting the highest biofilm formation, 4 × 3 and DH5 showing rapid auto-aggregation, 4 × 3 and ICS1 displaying high CSH, and all strains demonstrating considerable adherence to Caco-2 cells. 4 × 3 also exhibited exceptional bile tolerance, while ICS1 showed robust survival under simulated gastrointestinal conditions. These traits suggest *M. pulcherrima*’s capacity to colonize the poultry gastrointestinal tract, promote animal health, and support more sustainable livestock practices as a potential alternative to conventional antibiotics.

**Sustainability Statement**

Expanding on previous research that has demonstrated the potent antimicrobial effects of *Metschnikowia pulcherrima*, our study investigates its potential as a sustainable alternative to conventional antibiotics in animal agriculture. Our findings underscore promising probiotic traits in *M. pulcherrima*, hinting at its potential to colonize within the poultry gastrointestinal tract, promoting health and supporting more sustainable livestock practices. This work directly aligns with and advances the following United Nations (UN) sustainable development goals (SDGs):

1. **SDG 2: zero hunger–**this research directly contributes to more robust food production systems and supports food security by improving animal health and reducing the need for antibiotics.
2. **SDG 3: good health and well-being–**developing antibiotic alternatives is crucial for mitigating antimicrobial resistance and safeguarding animal and human health.

## Introduction

Currently, a substantial proportion of antibiotics produced are for animal agriculture, leading to a rise in antimicrobial resistance (AMR), and the overuse of antibiotics may deplete our arsenal of effective antibiotics (Van Boeckel et al. 2015). AMR is a persistent threat in every country. It is estimated to result in over 700 000 premature deaths yearly, a figure that is expected to rise to 10 million by 2050 if effective treatments cannot be found (O'Neill 2014). To address this problem, antibiotic alternatives in the food production industry are now being investigated, such as improved hygienic and biosecurity protocols, probiotics, and vaccination. In recent years, probiotic supplementation has been found to have a substantial positive effect on animals, helping not only enhance health but also increase animal productivity (Park and Kim 2014). Once inside the animal gut, probiotic organisms can directly combat pathogenic organisms via various mechanisms, including regulating pH, inhibiting pathogen adhesion, and producing antimicrobials and enzymes (Mcdonald et al. 2010). There has also been evidence that probiotics can improve the host's immune system via direct communication via toll-like receptors (TLRs), resulting in an immunomodulatory effect (Lebeer et al. 2010).

The World Health Organisation (WHO) and the Food and Agriculture Organisation of the UN (FAO) have defined a probiotic as a ‘live microorganism that, when administered in adequate amounts, confer a health benefit to the host (Group 2002). Therefore, an organism must withstand the low pH and high concentrations of bile and enzymes and then successfully colonize the microbiome to adhere to the epithelial cells of the gut.

Lactic acid bacteria (LAB) are the most commonly used probiotics within commercial farming practices, having already been found in the guts of healthy animals (Bhogoju and Nahashon 2022). Mechanistically, LABs produce lactic acid, creating an adverse environment for pathogenic bacteria and strengthening the mucosal barrier against pathogens (Bermudez-Brito et al. 2012). In contrast, yeasts are an emerging frontier in this field; supplementation of yeasts within monogastrics either comes in the form of derivatives (hydrolysate, culture, extracts and cell wall) the other form is live yeast, all of which have been promising (Sampath et al. 2021, Sampath et al. 2021, Sampath et al. 2023). However, yeast probiotics still make up a small fraction of probiotic research (Staniszewski and Kordowska-Wiater 2021). As eukaryotes, yeasts can also induce many of the same effects as LABs, and being much larger than bacteria results in more significant steric hindrance of pathogenic organisms. From this position, then can directly enhance the nutritional value of the animal's diet by introducing minerals and vitamins (Abid et al. 2022).

Among potential probiotic yeasts, *Metschnikowia pulcherrima* is a non-saccharomyces yeast found mostly on various flowers and fruits. Not only does *M. pulcherrima* flourish in the highly acidic environment of grape must, but it has also been shown to be a notable biocontrol agent exhibiting substantial antimicrobial effects against other microbes (Oro et al. 2014, Türkel et al. 2014). Antimicrobial effects are commonly attributed to the iron chelator pulcherriminic acid, which is also responsible for its red colour. However, other control mechanisms have also been reported, as *M. pulcherrima* has also been shown to produce the antimicrobial compound 2-Phenylethanol (2-PE) and various lytic enzymes capable of degrading cell walls and membranes of competing organisms, positioning it as an ideal probiotic candidate (Banani et al. 2015, Chantasuban et al. 2018, Bühlmann et al. 2021, Steglińska et al. 2022). Recently, there has been an increasing interest in exploring the probiotic potential of *M. pulcherrima*. These emerging research trends emphasise the potential of *M. pulcherrima* as a valuable industrial yeast and a promising probiotic (Cristina Vergara Alvarez et al. 2023, Staniszewski and Kordowska-Wiater 2023, Commenges et al. 2024).

Previous work has also shown that specific strains of *M. pulcherrim*a can inhibit the growth of industrially problematic organisms such as avian pathogenic *Escherichia coli* (APEC), *Salmonella* spp. and *Staphylococcus* spp. (Hicks et al. 2021). Suppression effects were not passive but instead were part of a distinct response to the presence of these microbes via the production of specific proteins. *M. pulcherrima* has been shown to metabolize a wide range of sugars and cultured under a minimally controlled environment and non-sterile conditions (Santamauro et al. 2014), and is an ideal biotechnological organism.

This study is the first of its kind to comprehensively evaluate the probiotic potential of *M. pulcherrima* strains for poultry probiotic applications. We assessed the previously reported strains ICS1 and QRI1 for their antimicrobial properties (Hicks et al. 2021), an evolved strain 4 × 3 designed for enhanced lipid production, and a high 2-PE producing strain DH5, assessing their biofilm production, auto-aggregation, cell-surface hydrophobicity, adhesion to Caco-2 epithelial cells, antioxidant capacity, and viability under simulated poultry gastric conditions. By evaluating these properties, we seek to provide insights into the suitability of *M. pulcherrima* as a novel poultry probiotic and its potential to serve as a sustainable alternative to antibiotics in animal agriculture.

## Materials and methods

### Yeast cultures

All strains (Table 1) used in this study were stored on sterile Malt extract agar (MEA) plates (Peptone from soybean, enzymatic digest 5 g/L; Oxoid™ Malt Extract 30 g/L; Agar 15 g/L, pH 5.5; in deionised water) at 4°C and refreshed monthly to retain viability.

**Table 1.** Overview of *M. pulcherrima* strains used in the study.

Strain ID	Source	Notes
4 × 3	Bath, UK	NCYC 4331, inhibitor tolerant strain, evolved from 2580 (Hicks et al. 2020).
DH5	Bath, UK	Low inhibitory activity against *S. aureus, Salmonella* and APEC (Hicks et al. 2021).
ICS1	Bath, UK	Antimicrobial activity against *S. aureus, Salmonella* and APEC (Hicks et al. 2021).
QRI1	Bath, UK	Antimicrobial activity against *S. aureus, Salmonella* and APEC (Hicks et al. 2021).

Detailed strains (4 × 3, DH5, ICS1, QRI1), origin (Bath, UK) and characteristics. Strain 4 × 3 evolved in Bath, noted for inhibitor tolerance; ICS1, DH5, and QRI1 all wildtype exhibiting antimicrobial activity against *S. aureus, S. enterica* and APEC *(E. coli)*.

Total viable counts (TVCs) were enumerated via serial dilution using phosphate-buffered saline (PBS) (Sodium chloride 80 g/L; Potassium chloride 2 g/L; Disodium phosphate 14.4 g/L, Monopotassium phosphate 2.4 g/L pH 7.4; in deionised water) and spreading 100 µl of sample on an MEA plate.

Yeast cultures were performed in 100 ml Erlenmeyer flasks with a foam stopper and filled with 25 ml of soybean malt extract broth (SMB) media (Peptone from soybean, enzymatic digest 30 g/L; Oxoid™ Malt Extract 25 g/L; pH 5).

### Biofilm formation

A Biofilm production assay was adapted from (Cristina Vergara Alvarez et al. 2023). Overnight cultures of *M. pulcherrima* were incubated at 25°C in SMB within 100 ml Erlenmeyer flasks. Cultures were then diluted to an OD of 0.1 (∼4.7 × 10^5^). For the assay, 200 µl of each diluted culture was transferred into eight wells of a 96-well plate (Corning™ Costar™ 96-Well). Plates were incubated for 24 hours at 25°C shaking at 200 RPM. SMB alone served as a blank control. Post-incubation, the yeast suspension was gently removed, and each well was rinsed twice with 200 µl of PBS, followed by air-drying to fix the biofilm onto the wells. Wells were stained with 200 µl of 1% crystal violet for 30 minutes at room temperature. After staining, wells were washed with distilled water to remove excess stain. Biofilm was then released by adding 200 µl of 96% ethanol and quantified by measuring optical density at 620 nm (OD620)—the positive difference in OD620 between yeast cultures and the SMB blank determined biofilm formation.

### Auto-aggregation

Auto-aggregation was performed as previously described, with modifications (Ogunremi et al. 2015). Overnight cultures of *M. pulcherrima* were grown in SMB (25°C, 200 RPM shaking). Cultures were centrifuged at 3000 RPM for 10 minutes at room temperature. The supernatant was discarded, and the cell pellets were re-suspended with 5 ml of sterile PBS and then incubated for auto-aggregation at 37°C. Samples were then gently taken from the upper suspension at 2,4, and 24 hours and measured for OD600. Auto-aggregation was calculated using equation 1, where A_0_ is the initial OD600, and A_t_ is the OD600 at the respective time points.


\begin{eqnarray*}
\mathrm{Auto} - \mathrm{aggregation}(\%) = 1 - \frac{{\mathrm{A}}_{\mathrm{t}}}{{\mathrm{A}}_{0}} \times 100.
\end{eqnarray*}

Equation 1: calculation of auto-aggregation where A_0_ represents initial OD600 at time 0 and A_t_ represents OD600 at 2, 4, and 24 hours.

### Hydrophobicity

A modified method, as described previously, was used to measure the hydrophobicity of *M. pulcherrima* strains (Fadda et al. 2017). Overnight cultures grown in SMB (25°C, 200 RPM shaking) were centrifuged at 3000 RPM for 10 minutes, adjusted, washed with PBS, and diluted to an OD600 of 1.

For the assay, 3 ml of these yeast suspensions were added to 0.6 ml of xylene (Thermo Fischer Scientific 10 385 910), and samples were then vortexed for 120s to form an emulsion. Samples were then incubated for 1 hour at 37°C for phase separation. The bottom aqueous phase was carefully removed. Hydrophobicity was calculated based on the reduction in OD600 from the initial yeast suspension (OD_0_) to that of the aqueous phase after incubation (OD_aq_).


\begin{eqnarray*}
{\mathrm{Hydrophobicity\ }}\left( {\mathrm{\% }} \right) = \frac{{{\mathrm{O}}{{{\mathrm{D}}}_0} - {\mathrm{O}}{{{\mathrm{D}}}_{{\mathrm{aq}}}}}}{{{\mathrm{O}}{{{\mathrm{D}}}_0}}} \times 100.
\end{eqnarray*}

Equation 2: calculation of cell surface hydrophobicity (CSH) in xylene where OD_0_ represents the initial OD600 of the yeast suspension and OD_aq_ is the OD600 of the aqueous phase post-separation in xylene after 1 hour.

### Adhesion

A modified protocol was used to assess the adhesion of *M. pulcherrima* to Caco-2 cells (Živković et al. 2015). *M. pulcherrima* strains were cultured at 25°C for 48 hours, shaking at 200 RPM within SMB media in 100 ml Erlenmeyer flasks. Post-culture yeast cells were centrifuged, washed with PBS, and resuspended to a concentration of 10^8^ CFU/ml. Caco-2 cells, passaged from a T75 flask, were cultured in a 24-well plate (1.9 cm^2^/well) using high glucose Dulbecco's Modified Eagle Medium (DMEM; Sigma–Aldrich DH5796) supplemented with 10% fetal bovine serum (FBS; Thermo Fischer Scientific 10 270 106). Incubation was at 37°C and 5% CO_2_ until ∼95% confluency was reached. Cell counts were determined to be 2 × 10^5^ cells/well via four sacrificial wells using a haemocytometer (0.100 mm depth, 0.0025 mm^2^; Marienfeld Germany). After washing monolayers with pre-warmed PBS (PBS; Thermo Fisher Scientific 10 010 023), yeast cells were added at a multiplicity of infection (MOI) of 10:1 (*M. pulcherrima*: Caco-2). Adhesion experiments were conducted in duplicate and incubated for 1 hour at 37°C. The monolayers were gently washed with PBS, and MEA plating was performed to determine unadhered yeast cells. 0.25% trypsin was added for 5 minutes at 37°C to detach adhered cells, which were also plated.


\begin{eqnarray*}
{\mathrm{Adhesion\ }}\left( {\mathrm{\% }} \right) = \frac{{{\mathrm{adhered\ yeast\ CFU}}}}{{{\mathrm{Adhered\ Yeast}} + {\mathrm{Unadhered\ Yeast}}}} \times 100.
\end{eqnarray*}

Equation 3: calculation of *M. pulcherrima* adhesion to Caco-2, where total yeast CFU is the sum of adhered and unadhered yeast CFUs, determined from duplicate MEA plates.

### Bile tolerance

Bile tolerance of *M. pulcherrima* using a modified protocol as described previously was used (Prete et al. 2020). Overnight cultures were grown in SMB at 25°C with shaking at 200 RPM in 100 ml Erlenmeyer flasks. The cultures were passaged into triplicate flasks, each starting at an OD600 of 0.1. This was achieved by diluting overnight cultures in fresh media containing varying concentrations of ox-gall (Bile bovine powder; Thermo Fischer Scientific 460 330 250), ranging from 0% to 1.5% (w/v). The growth of the yeast, as indicated by changes in OD600, was monitored twice daily, providing an understanding of the ability of the strain to tolerate bile.

### Antioxidant activity

Antioxidant capacity and Trolox equivalence of strains were assessed as described initially (CHEN et al. 2010), with the appropriate modifications (Menezes et al. 2020). Cultures were grown for 48 hour within borosilicate tubes in SMB (as detailed previously) media at 25ºC and 200 RPM. To ensure standardized cell density, cultures were diluted to an OD600 of 10 in a 1 ml volume using 0.9% NaCl. Samples underwent centrifugation (5 minutes at 12 000 RPM), followed by two washes using an equal volume of 0.9% NaCl. Washed cells were re-suspended in the same volume of 0.9% NaCl.

For the antioxidant assay, 800 μl of the cell suspension was added to a 1 ml DPPH (2,2-Diphenyl-1-picrylhydrazyl, Sigma–Aldrich D9132-1 G) solution (0.2 mM methanol) into a new tube. This mixture was incubated for 30 minutes in darkness, then transferred into an Eppendorf tube and centrifuged for 5 minutes at 9000 RPM. Subsequently, 300 μl of the supernatant was carefully transferred into a 96-well plate (Corning™ Costar™ 96-Well). to measure the absorbance at 517 nm. Trolox ((±)-6-Hydroxy-2,5,7,8-tetramethylchromane-2-carboxylic acid; Sigma–Aldrich 238 813) standards were made from 10–100 µmol/ml to assess Trolox equivalence, calibration curve (y=-0.134x + 1.31) with a linearity of R^2^ = 0.95 was used.


\begin{eqnarray*}
{\mathrm{Antioxidant}}\ \left( \% \right) = \frac{{1 - {\mathrm{Sampl}}{{{\mathrm{e}}}_{{\mathrm{\ Abs\ }}517{\mathrm{nm}}}}}}{{{\mathrm{Blan}}{{{\mathrm{k}}}_{{\mathrm{\ Abs\ }}517{\mathrm{nm}}}}}}\ \times 100.
\end{eqnarray*}

Equation 4: percentage of antioxidant capacity is determined by comparing the absorbance of the sample ($\bf{Sample}_{\bf {Abs}}\ 517{{\bf nm}}$) with the absorbance of the blank ($\bf {Blank}_{\bf {Abs}\ 517{\bf {nm}}}$). As measured by the plate reader (blank uncorrected).

### Gastrointestinal enzyme and low pH tolerance

The gastrointestinal tolerance of *M. pulcherrima* was assessed using a modified *in vitro* assay. (Sommerfeld et al. 2017). A 100 ml Erlenmeyer flask containing 25 ml SMB media was inoculated and then incubated at 25°C and 170 RPM, shaking for 48 hours. Pepsin stock solution was prepared by dissolving pepsin (pepsin from porcine; Sigma–Aldrich 77 160) in 1.5 M HCl to the desired concentration. Similarly, pancreatin (pancreatin from porcine, 8x IU; Sigma–Alrich P7545) was dissolved in 1 M Na₂CO₃ to create the base solution. Two autoclaved 100 ml two-neck round bottom flasks (RBF) containing a magnetic stir bar were placed in a 40°C water bath for the assay. Then, 50 ml of sterile PBS was added to the flasks and continuously stirred.

**Figure 1.**
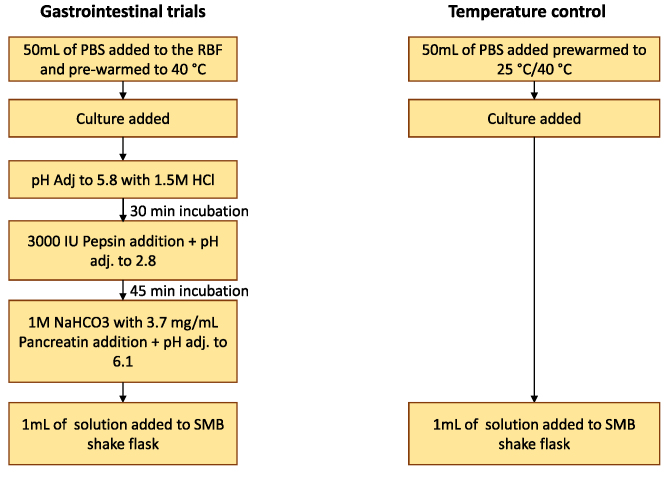
The gastrointestinal experiment process flow modified from (Sommerfeld et al. 2017), illustrates the steps mimicking the pH changes and enzymatic digestion within a broiler's GIT, including the addition of a yeast culture, pH adjustments and incubation times.

*M. pulcherrima* was added to the PBS to a final concentration of ∼2.2 × 10^7^ CFU/ml. The subsequent experimental steps, including pH adjustments and enzymatic treatments to simulate a broiler's gastrointestinal tract (GIT), were formed as illustrated in Fig. 1. To determine yeast survival, samples were collected at various time points during the assay, serially diluted using PBS, and then plated on MEA plates. The plates were incubated at 25°C for 48 hours, and the colony-forming units (CFU) were enumerated to calculate the survival of *M. pulcherrima* under simulated gastrointestinal conditions.

### Data analysis

All data were processed, and graphs were generated using Microsoft Excel. Statistical analysis was performed using one-way ANOVA followed by Tukey's HSD post-hoc test to identify significant differences (*P* < 0.05) among *M. pulcherrima strains* for the assessed probiotic properties. In figures, strains with different letters above the bars indicate differences based on the post-hoc test.

## Results

### Biofilm production

**Figure 2.**
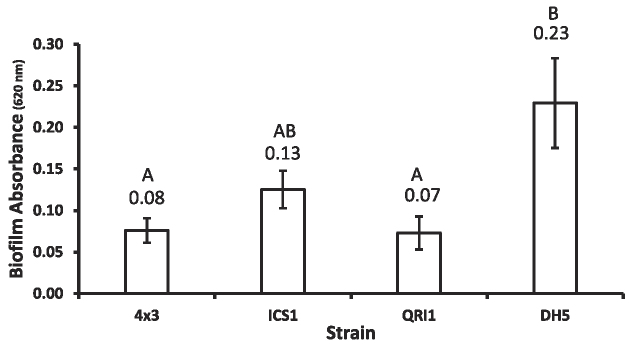
Comparative biofilm production of different *M. pulcherrima* strains. The graph shows the mean biofilm absorbance at 620 nm for strains 4 × 3, ICS1, QRI1, and DH5 after 24 hours of incubation in SMB within a 96-well plate. Error bars represent propagated SE. Strains with different letters above the bars indicate significant differences in biofilm production (*P* < 0.05, Tukey HSD post-hoc test).

Biofilm formation was quantitatively assessed among various probiotic strains over 24 hours in SMB medium, using a 96-well plate assay with absorbance measured at 620 nm. The results indicated a significant variance in biofilm production capabilities across the strains. Specifically, the DH5 strain demonstrated notably higher biofilm production, achieving an absorbance of 0.23. This was significantly higher than the other strains tested (Fig. 2). In contrast, the biofilm production of the 4 × 3 strain was assessed. Despite a propensity for high CSH and rapid auto-aggregation, it exhibited the lowest biofilm formation among the tested strains, with an absorbance of 0.08.

### Auto-aggregation

**Figure 3.**
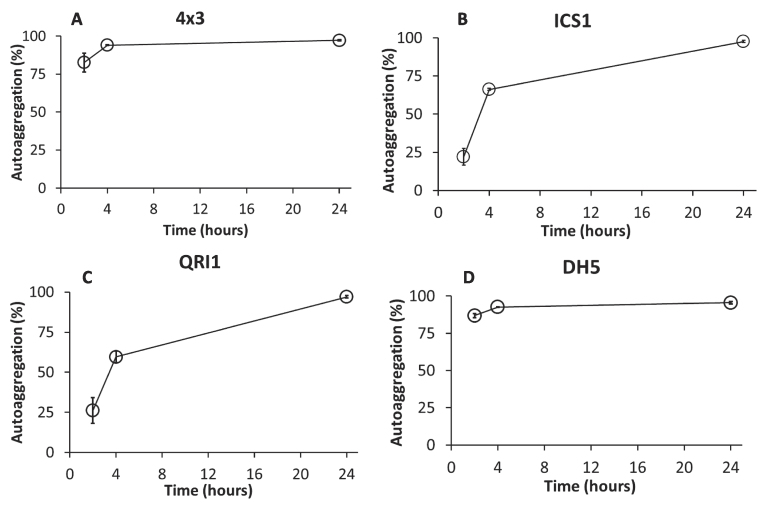
Time-dependant auto-aggregation ability of *M. pulcherrima* strains (4 × 3, ICS1, QRI1, and DH5) in PBS over 24 hours. Error bars represent the SEM.

Auto-aggregation, a property indicative of a probiotic's potential for robust gut epithelial adhesion and survival in harsh conditions, was assessed among *M. pulcherrima* strains. All strains could auto-aggregate over 24 hours at 37°C in PBS, as shown in Fig. 3.

4 × 3 and DH5 strains showed the most pronounced early auto-aggregation, reaching above 80% within the initial hour and maintaining that level throughout the period. Conversely, ICS1 and QRI1 displayed a more incremental rise in auto-aggregation over the same period.

### Hydrophobicity

CSH of *M. pulcherrima* strains was evaluated for their affinity to hydrophobic substrates. Xylene was employed as the hydrophobic phase to assess CSH—a known proxy for the adherence potential of probiotic strains to gut epithelial cells.

**Figure 4.**
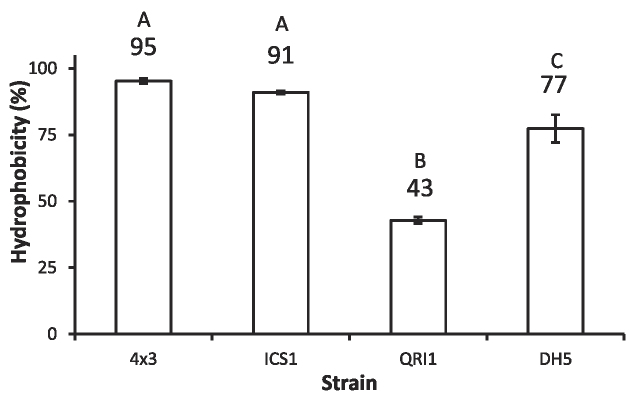
CSH of *M. pulcherrima* strains. The graph represents different CSH, quantified as the percentage of yeast culture remaining in the xylene phase, determined by optical density at 600 nm after 1 hour. Error bars represent propagated SE. Strains with letters above the bars indicate differences in hydrophobicity production (*P* < 0.05, Tukey HSD post-hoc test).

The assay revealed significant differences in CSH among the strains. Strains 4 × 3 and ICS1 displayed exceptionally high levels of CSH, with 4 × 3 at 95%, indicative of its oleaginous nature (Fig. 4) . The QRI1 strain demonstrated markedly lower CSH compared to the other strains. DH5 exhibited a moderate level of hydrophobicity, higher than QRI1 but lower than 4 × 3 and ICS1.

### Adhesion

**Figure 5.**
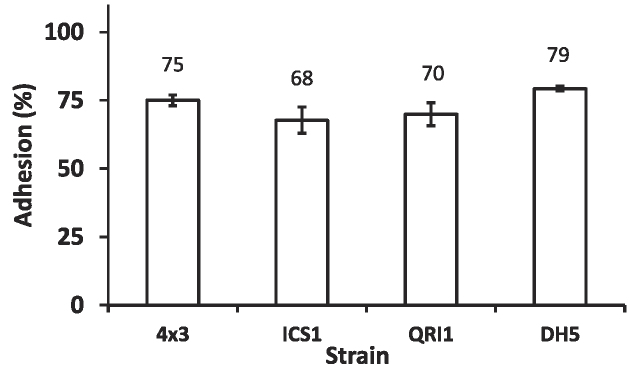
Graph represents the adhesion capacity of different *M. pulcherrima* strains to Caco-2 cells, measured by calculating the percentage of viable adherent cells relative to the initial cell count. Error bars represent SEM. No significant differences were observed in adherence (*P* > 0.05, Tukey HSD post-hoc test).

The ability of *M. pulcherrima* strains to adhere to epithelial cell walls, an essential factor for gut colonization, was assessed using the Caco-2 cell line as a model. All strains demonstrated considerable adherence capabilities (Fig. 5).

Although DH5 displayed the highest adherence percentage, statistical analysis revealed no significant differences in the adhesion rates among the strains test (*P* > 0.005, Tukey HSD post-hoc test).

### Bile tolerance

The resistance of *M. pulcherrima* strains to bile was evaluated as an indicator of their ability to survive in the GIT. Strains were cultured in SMB broth supplemented with varying concentrations of oxgall, from 0% to 1.5% (w/v), to mimic gut conditions.

**Figure 6.**
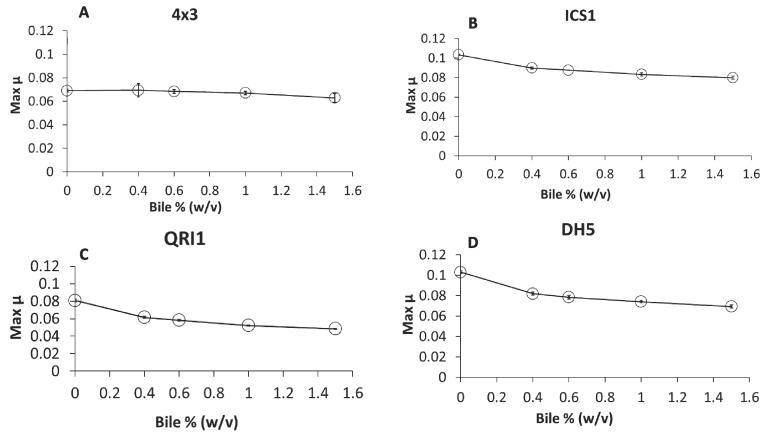
Maximum growth rates of *M. pulcherrima* strains (A: 4 × 3, B: ICS1, C: QRI1, D: DH5) in response to varying ox gall concentrations. Values are calculated from OD600 taken from triplicate shake flasks. Error bars represent SEM.

In this assay, all strains demonstrated the ability to grow in the presence of 1.5% (w/v) oxgall, showcasing strong resistance to bile, a key factor for survival and function within the GIT (Fig. 6).

**Figure 7.**
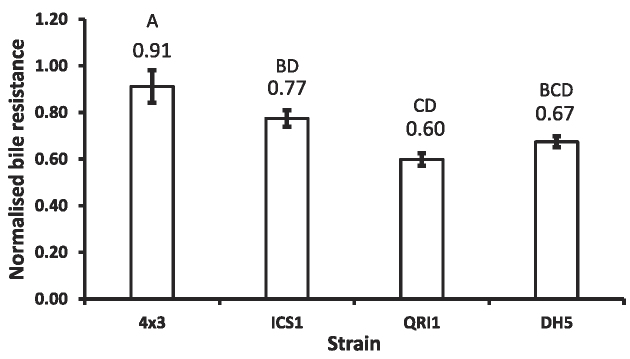
Normalized bile resistance of *M. pulcherrima* strains. The bar chart shows the normalized maximum growth rates of four *M. pulcherrima* strains (4 × 3, ICS1, QRI1, DH5) when cultured with SMB broth). Values represent the growth rate ratio in the presence of broth containing 1.5% oxgall (w/v) to the control growth rate without oxgall. Error bars denote the mean (SEM) standard error from triplicate experiments. Strains with letters above the bars indicate signifcant differences in normalised bile resistance (*P* < 0.05, Tukey HSD post-hoc test).

The normalised bile resistance was calculated for each strain, with all strains demonstrating the ability to grow at the highest concentration of oxgall tested (Fig. 7). Strain 4 × 3 showed remarkable resilience, retaining 91% of its maximum growth rate at the highest oxgall concentration tested. The other strains, while slightly affected by the presence of oxgall, also exhibited considerable growth. This suggests that these strains possess the potential to withstand the harsh conditions of the gastrointestinal environment.

### Antioxidant activity

**Figure 8.**
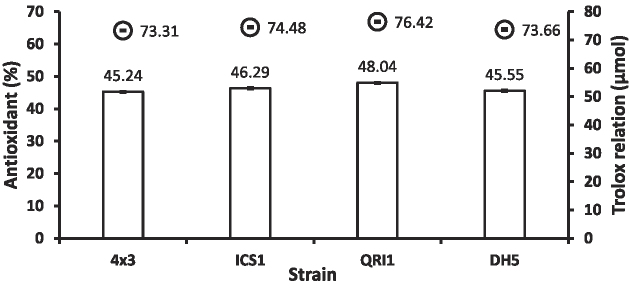
Comparative antioxidant activity and Trolox Equivalence of *M. pulcherrima* strains: Bars represent (%) of DPPH radical scavenging capacity, indicating antioxidant activity, for each of the four strains test: 4 × 3, ICS1, Q1, and DH5. The circles overlaying the bars represent each strain's Trolox equivalent antioxidant capacity (in µmol). Datapoints represent mean values from triplicate readings. No significant levels were observed in antioxidant levels among the strains (*P* > 0.05, Tukey post-hoc test)

This study assessed the antioxidant activities of four strains of *M. pulcherrima*, comparing them against the established antioxidant benchmark, Trolox (vitamin E analogue). Our quantitative analysis showed a narrow range of DPPH scavenging abilities of all strains lay between 45.24% and 48.04%, with the evolved strain 4 × 3 displaying the lowest antioxidant capacity (45.24%); there was no statistically significant difference between any of the strains tested (*P* < 0.05, Tukey post-hoc test). A Trolox equivalent was also calculated for each strain, as displayed in Fig. 8.

### Simulated poultry gastrointestinal survival of ICS1

The viability of *M. pulcherrima* strain ICS1 was evaluated under simulated poultry GIT conditions. ICS1 was chosen due to its proven antimicrobial properties and promising probiotic traits.

A modified tailored environment was employed to mimic the GIT, incorporating a pH range from 2.8 to 6.1, digestive enzymes (pepsin and pancreatin), and an elevated temperature of 40°C.

**Figure 9.**
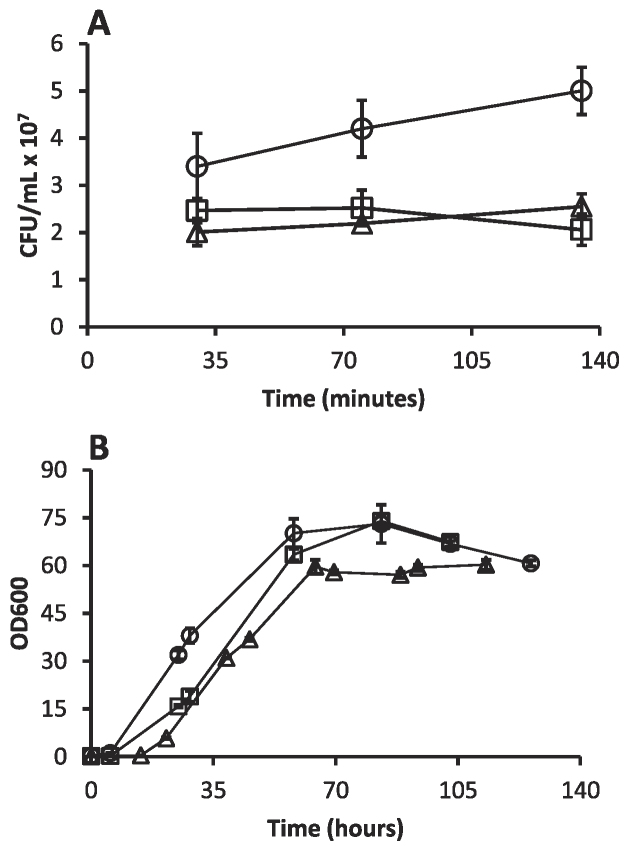
(A) Viability of ICS1 during the simulated gastric pass under various conditions. Triangles represent 40°C + GIT conditions, circles represent 40°C + PBS conditions, and squares represent 25°C + PBS conditions. TVC data points for room temperature plots based on two plate replicates and GIT conditions based on 4 plate replicates. (B) Post gastric simulation culturability of ICS1, data presented from 3 triplicate shake flasks for each condition.

Figure 9A depicts ICS1’s tolerance to varying pH levels and enzymatic pressures. At room temperature in PBS, ICS1 exhibited minimal viability reduction across the tested pH range of 2.8–6.1. However, when subjected to simulated GIT conditions at 40°C, a marked decrease in survivability was observed. Following gastric transit, ICS1 demonstrated preserved culturability in SMB, as shown in Fig. 9B.

## Discussion

The present study provides a comprehensive assessment of the probiotic potential of *M. pulcherrima* strains, focusing on their suitability for poultry gastrointestinal environments. The results demonstrate that the tested strains exhibit promising probiotic traits, suggesting their potential as alternatives to conventional antibiotics in agriculture. This study comprehensively assesses the probiotic potential of *M. pulcherrima*, particularly in the context of poultry agriculture. The tested strains exhibited a range of desirable probiotic traits, suggesting their potential as alternatives to antibiotics in poultry feed.

Biofilm formation is crucial to gut colonization and interaction within the host environments. While previously associated with virulent strains (Bjarnsholt 2013, Barzegari et al. 2020). Recent evidence suggests that biofilm formation is a conserved trait for an organism's survival in hostile environments, and many commensal microbes are biofilm producers (Hancock et al. 2010). Probiotics capable of producing biofilms can defend against competing and pathogenic organisms, aid in gut colonization, and prevent adhesion of other organisms (Salas-Jara et al. 2016). Furthermore, biofilm cells have been shown to enhance metabolic interplay within the gastrointestinal ecosystem and exert positive immunomodulatory effects compared to planktonic cells (Liu et al. 2023). The tested *M. pulcherrima* strains showed varying levels of biofilm production, with the DH5 strain exhibiting significantly higher biofilm formation, potentially due to its heightened ability to produce quorum-sensing molecules, in particular, 2-phenylethanol (Zhang et al. 2021). This suggests DH5’s robust capacity for gut colonization. Notably, the widely used yeast probiotic *Saccharomyces cerevisiae* var. *boulardii* is a “poor biofilm producer”, resulting in 0.025 absorbance units (AUs) under similar conditions. (Cristina Vergara Alvarez et al. 2023), further highlighting the superior biofilm-forming capabilities of the *M. pulcherrima* strains we tested.

Auto-aggregation and CSH are key indicators of the ability of a probiotic to adhere to gut epithelial cells and survive harsh environments (Rahman et al. 2008, Sourabh et al. 2011). However, in this respect, both indicators are independent (Rahman et al. 2008, Sourabh et al. 2011). The tested strains within this study demonstrated rapid auto-aggregation, with 4 × 3 and DH5 exceeding 75% auto-aggregation within 2 hours. The variability at the earlier time points across *M. pulcherrima* strains has also been noted previously (Bonatsou et al. 2018). Regarding hydrophobicity, we also show how strains 4 × 3 and ICS1 exhibit superior CSH compared to other yeasts such as *K. lactis, K. marxianus*, and *S. cerevisiae* (Fadda et al. 2017). The oleaginous strain 4 × 3 showed the highest CSH, which may be attributed to its oleaginous characteristics, as observed in *Y. lipolytica* (Thevenieau et al. 2010). The high auto-aggregative ability of 4 × 3 and DH5 correlated with their adhesion to Caco-2 monolayers, despite the difference in hydrophobicity demonstrating how different probiotic indicators influence adhesion and reaffirming their potential for gut colonization.

Adhesion to epithelial cells is crucial for probiotics to colonize the gut and confer their beneficial effects. All *M. pulcherrima* strains demonstrated considerable adherence to Caco-2 cells. DH5 had the highest adherence at 79%, while ICS1 had the lowest at 68%.

These adhesion rates are relatively high, especially compared to the range of 30–75% observed for various yeast species in a similar assay (Živković et al. 2015), placing our *M. pulcherrima* strains at the higher end of the adherence scale. Similar protective properties against epithelial cell barrier disruption have also been observed in other *Metschnikowia* species (Smith et al. 2015), suggesting that such traits may extend across the genus.

Bile tolerance is a critical factor determining probiotic efficacy in the GIT. The tested *M. pulcherrima* strains showed remarkable resilience to bile, with strain 4 × 3 exhibiting exceptional bile tolerance, retaining 91% of its maximum growth rate in the presence of 1.5% oxgall. This finding is noteworthy, as other probiotic yeasts have shown declining cell counts at lower concentrations (Psomas et al. 2001), whereas we show growth (albeit reduced). The tenacity of 4 × 3 against oxgall may be an extension of its evolved resistance to various inhibitors (Hicks et al. 2020).

Antioxidant activity is another desirable trait in probiotics, contributing to host health and improving the oxidative stability of animal products. The tested strains exhibited robust DPPH scavenging abilities, falling within the ‘very good activity’ category for DPPH radical scavenging (Gil-Rodríguez et al. 2015). The uniformity of antioxidant activity observed in *M. pulcherrima*, as opposed to the broader variability in *S. cerevisiae* (Gil-Rodríguez et al. 2015), highlights the potential of *M. pulcherrima* as a species to enhance host health and promote oxidative stability of meat products (Zhang et al. 2005). The exceptionally high antioxidant scavenging capacity of *M. pulcherrima* is also in line with a previous study, where they found an even higher range from 60% to 70% (Staniszewski and Kordowska-Wiater 2023), which may be attributed to the ability of *M. pulcherrima* to produce pulcherrimin (Jayalakshmi et al. 2012), a known antioxidant.

The survivability of probiotic strains in the GIT is crucial for evaluating their efficacy. The poultry GIT presents a complex environment with varying pH, temperature, and enzyme activity that can significantly affect probiotic viability (Scanes and Pierzchala-Koziec 2014). ICS1, selected for its robust performance and demonstrated antimicrobial effects (Hicks et al. 2021), showed tolerance to varying pH and enzymatic conditions in an environment mimicking the poultry GIT. Although its survivability (as assessed by TVCs) was reduced at elevated temperatures, it was able to grow within highly acidic environments, which is in line with previous research (Santamauro et al. 2014). Furthermore, ICS1 maintained culturability post-gastric transit, demonstrating its robustness to survive the initial harsh environment and thrive as a probiotic. This data correlates with another study showing excellent survival rates of *M. pulcherrima* in a human gastrointestinal assay (Staniszewski and Kordowska-Wiater 2023), suggesting that this grit may be inherent to the species rather than strain-specific. The importance of yeast colonization in the poultry GIT should not be overlooked. The mycobiome in poultry primarily resides in the crop and ventriculus, with yeasts struggling to penetrate lower regions like the duodenum due to enzymes such as trypsin (Scanes and Pierzchala-Koziec 2014, Fathima et al. 2023). This highlights the significance of ICS1’s performance in the simulated GIT conditions, as its ability to survive and maintain culturability in this challenging environment is a critical factor in its potential as a probiotic for poultry.

In conclusion, this study highlights the promising probiotic potential of *M. pulcherrima* strains for poultry applications. The tested strains, particularly ICS1, demonstrated robust survival under simulated poultry GIT conditions, strong adhesion properties, and desirable antioxidant activity. Future research should aim to confirm these capabilities *in vitro* findings within live poultry models and assess their impact on animal health, productivity, and overall sustainability of poultry production practices.

## Data Availability

The data underlying this article will be shared on reasonable request to the corresponding author.
